# Exploring perceptions of consanguineous unions with women from an East London community: analysis of discussion groups

**DOI:** 10.1007/s12687-019-00429-4

**Published:** 2019-07-16

**Authors:** Meghan A. Cupp, Mary Adams, Michelle Heys, Monica Lakhanpaul, Emma C. Alexander, Yasmin Milner, Tausif Huq, Meradin Peachey, Lakmini Shah, Iram Shazia Mirza, Logan Manikam

**Affiliations:** 1 UCL Great Ormond Street Institute of Child Health, 30 Guilford Street, London, WC1N 1DP UK; 2 Aceso Global Health Consultants Limited, 3 Abbey Terrace, London, SE2 9EY UK; 3 King’s College London, Division of Women and Children’s Health, Faculty of Life Science and Medicine, St Thomas’ Hospital, London, SE1 7HE UK; 4 East London NHS Foundation Trust, Trust Headquarters, 9 Alie Street, London, E1 8DE UK; 5 Whittington NHS Trust, Magdala Avenue, London, N19 5NF UK; 6 King’s College London School of Medical Education, Hodgkin Bldg, Newcomen St, London, SE1 1UL UK; 7 UCL Institute of Epidemiology & Health, 1-19 Torrington Place, London, WC1E 7HB UK; 8 Newham University Hospital, Barts NHS Trust, 30 Guilford Street, London, E13 8SL UK; 9 London Borough of Newham, Newham Dockside, 1000 Dockside Road, London, E16 2QU UK

**Keywords:** Consanguinity, Genetic literacy

## Abstract

Consanguineous unions are relationships between blood relatives. This study explores the perceptions of consanguineous unions and risk of childhood disability and illness through the reported views and experiences of women in an ethnically diverse London community. This qualitative study utilised group discussions to elicit women’s views and experiences. Field notes were recorded by independent note-takers in four group discussions. Field notes were coded manually and independently by two researchers who identified common themes for thematic analysis. Thirty-six women attended, of whom 20 identified as Asian Pakistani. Identified themes included variation in participants’ views of consanguineous unions and associated health risks, the value of informed decisions and preferences for information distribution. Although participants had diverse opinions and experiences, they considered risk awareness to be vital for encouraging informed decisions in younger generations. This study highlights the importance of involving the community in efforts to increase awareness around consanguineous unions and genetic risk, emphasising the need for enabling educated choices and the value of co-developing educational efforts with the community.

**Electronic supplementary material:**

The online version of this article (10.1007/s12687-019-00429-4) contains supplementary material, which is available to authorized users.

## Background

Clinical genetics considers a relationship between blood relatives who are second cousins or closer as consanguineous (Hamamy et al. 2011; Ng 2016). Consanguinity describes the state of being related by blood and the terms consanguineous relationships or unions are used to describe relationships between blood relatives. Consanguineous unions are prevalent in many communities worldwide and it is estimated that, globally, 15% of all neonates have consanguineous parents (Bennett et al. 2002; Bittles and Black 2010; Darr 2016). In recent years, migration has led to increasingly multi-ethnic societies with a cultural milieu of diverse traditions and social norms. This has contributed to the spread of global awareness of the genetic implications of customary consanguineous marriages (Bennett et al. 2002; Modell and Darr 2002).

Consanguineous marriages have been linked to genetic disease due to an increased risk of autosomal recessive disorders and infant mortality (Bennett et al. 2002; Modell and Darr 2002; Hamamy 2012). Evidence suggests that the risk of inheriting a genetic disorder is doubled in the children of consanguineous parents, compared to children of unrelated parents (Bennett et al. 2002; Shaw 2009; Hamamy 2012; Darr et al. 2013). Congenital birth defects, such as sensorineural hearing loss and heart disease, and neurodevelopmental disorders, such as autism spectrum disorder and unexplained learning difficulty, also seem to occur in children born to consanguineous parents at high rates (Lyons et al. 2009; Strømme et al. 2009; Shieh et al. 2012; Ng 2016; Al-Mubarak 2017; Best et al. 2017; Sanyelbhaa et al. 2018). Although these disorders often have a complex aetiology which cannot be directly linked to genetics alone, this phenomenon can be partly explained by the increased likelihood of inheriting two recessive alleles, and hence manifestation of genetic disease (Modell and Darr 2002). Despite the potential health risks, consanguineous marriage is favoured in some populations due to social, cultural, and economic benefits, including the strengthening of family ties, confidence in finding a compatible spouse, and protecting property (Khlat et al. 1986; Bittles et al. 1991; Bittles 1994; Hussain 1999; Modell 2002; Khan et al. 2011).

In the United Kingdom (UK), studies involving families of Pakistani descent indicate higher rates of consanguineous unions (Darr 2016) and a threefold increase in child mortality when compared to Caucasians (Bundey and Alam 1993; Khan 2010). A cohort study in the London Borough of Tower Hamlets found a significantly increased risk of autosomal recessive disorders in children of consanguineous parents (33.6% versus 21.6%, *p* value = 0.011) (Best et al. 2017). Since the demographics of Tower Hamlets closely resembles the neighbouring borough of Newham (Office for National Statistics 2016), the results of this study raised awareness of the need to develop a public health response to address the needs of consanguineous families and marginalised communities in Newham. The UK has not established nation-wide action to support these families and only local approaches have been documented to date. These documented local approaches emphasise the importance of community engagement and co-design, to ensure that they are respectful of local beliefs and can be effectively implemented in the community (Salway et al. 2016; Ali et al. 2018). To this end, the present study sought to explore perceptions of consanguineous unions in a district of Newham and contribute to the growing body of evidence on local initiatives.

## Aims

The primary aim of the study was to explore perceptions of consanguineous unions and associated genetic risks, indirectly assessing genetic literacy at the community level. The secondary aim of the study was to examine proof of concept for future collaborative interventions involving genetic literacy in marginalised communities, such as ethnic minorities.

## Methods

Qualitative research, using facilitated small group discussion, was identified as the most suitable method for investigating community perceptions. This method combines interview-style questioning with group interactions to explore opinions, beliefs and experiences within a supportive and social framework whilst allowing researchers to observed shared language and knowledge within a group (Hughes and DuMont 1993; Krueger 1994). The ongoing conversation café initiative in Newham (Newham London n.d.) aims to engage, empower, and develop women and families in the community, presenting a good opportunity for hosting these group discussions. Topic guides were developed with input from a female community facilitator in order to structure group discussions (Table 1).

**Table 1:** Topic guide for focus group sessions

Topic guide for the focus group sessions	
1. To start, would you all like to share what has brought you here today?	
2. I have a few questions about the presentation:	
a. What do you think of the presented information?	
b. Did you know about this topic before today?	
c. How would you describe the awareness of this topic in your community?	
3. I would like to ask you more questions about your community.	
a. What would you think about a family member marrying a cousin?	
b. How would you describe the knowledge of consanguinity in your community?	
4. We are going to talk about the health problems associated with consanguineous marriage.	
a. What have you been told about these risks?	
b. What are couples entering a consanguineous marriage told about these risks?	
5. I am going to ask more questions about the link between consanguinity and health problems in children.	
a. What sources would you trust when learning about consanguinity?	
b. What do you think influences people’s beliefs?	
c. In your opinion, what is the role of your local borough in providing information on consanguinity?	
6. Imagine that you are part of the team responsible for sharing information with the community in Newham.	
a. What approach would you take?	
b. What are the key messages that need to be highlighted in the community?	
c. Who is the most important target for this information?	
d. What will people think of this information?	
7. Do you think information on consanguinity should be available to the wider community?	
a. Who do you see as the target for this?	
8. What do you think of children learning about consanguinity and genetics in school?	
9. Of everything that we discussed today, what do you think is the most important?	

### Recruitment of participants

Participants were recruited by the community facilitator using snowballing through purposive sampling for gender and ethnicity, focusing on females of South Asian and Middle Eastern descent. Potential participants were approached in street talks, a ladies’ Arabic group session, local libraries, schools, mosques and beauty parlours in Newham, London. Approximately 200 women were approached by the community facilitator, with 36 participants ultimately attending. Participants were not asked about their own marriage or relationship status for recruitment purposes or during the discussion groups to avoid stigmatisation. Demographic data and reasons for non-participation for those who did not agree to attend were not collected.

### Group discussions

Following a brief presentation on genetic disease in children of consanguineous parents and explanation of the study aims, participants were divided into four sub-groups of nine participants (Morgan 1997), each coordinated by an independent female facilitator. The discussions were hosted at East Ham Town Hall on 11/09/2017 and lasted for 90 min, with a 30-min-catered lunch break following question 5 (Table 1).

The facilitators were impartial mediators recruited from outside the local community to reduce any bias caused by pre-existing knowledge of community perceptions. All four facilitators were female and three of these facilitators had additional language skills, allowing for translations in Urdu, Hindi, Malayalam, Punjabi and Bengali. Ultimately, all participants felt comfortable carrying out discussions in English. Each facilitator guided discussion by offering prompts based on a topic guide (Table 1).

### Data collection

Data collection was conducted by each facilitator as field notes on a laptop, with verbatim quotations where possible. Voice recorders were not utilised for this data collection to encourage candidness, following advice from the community facilitator.

Demographic data on participants were collected using a questionnaire piloted by authors (Manikam et al. 2016). Confidentiality was maintained through coding of participant responses with assigned numbers corresponding to their anonymised demographic information.

### Data analysis

Responses from transcripts were reviewed and coded independently by MAC and MA to derive common themes and subthemes from the data through subsequent thematic analysis. Conflicts in data analysis were resolved by discussion with EA. In this study, we use thematic analysis to understand fundamental themes and their relationships within the participant group, including the range of individual attitudes, opinions and beliefs expressed (Guest et al. 2010; Bowling 2014).

## Results

### Participant characteristics

Of the 36 women included in the discussion groups, most (47%, *n* = 17) were between 30 and 39 years of age (Table 2) with a mean age of 39 years. The majority of participants were of Asian Pakistani descent (55%, *n* = 20), with the most common birthplace of participants being Pakistan (42%, *n* = 15), followed by the UK (25%, *n* = 9). Urdu was the most common native language (39%, *n* = 14) and Islam was the most commonly reported faith (97%, *n* = 35). The majority of participants (67%, *n* = 24) had lived in the UK for over 10 years, and all but two participants had children.

**Table 2:** Demographic information on participants

	Sample (*N* = 1X)	
Characteristic	*n*	%
Gender		
Female	36	100
Male	0	0
Age bands^♱^		
15–29 years^*^	2	6
30–44 years	26	72
45–59 years	5	14
60 years and over	2	6
Birth place		
Pakistan	15	42
United Kingdom	9	25
India	1	3
Bangladesh	4	11
Germany	1	3
South Africa	2	6
Iran	1	3
Afghanistan	3	8
Years living in the UK		
5–19 years	21	58
20–34 years	7	19
35–50 years	8	22
Religion		
Islam	35	97
Hindu	1	3
Native Language^♱^		
Urdu	14	39
Gujrati	5	14
Tamil	1	3
Punjabi	3	8
Hindi	2	6
English	1	3
Bengali	4	11
Arabic	2	6
Kurdish	1	3
Farsi Dari	1	3
Persian	1	3
Ethnic group		
Asian Pakistani	20	56
Asian Indian	6	17
Sri Lankan	1	3
Asian Bangladeshi	4	11
British Algerian	2	6
Afghan	1	3
Other Asian	1	3
Persian	1	3

^♱^one participant omitted their response, *only 2 participants were aged 19 years

### Emergent themes

**Fig. 1:**
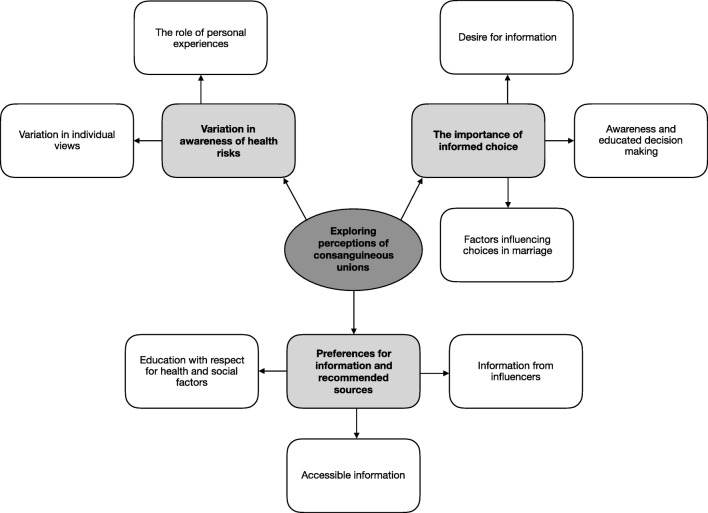
Concept map of emergent themes from thematic analysis of discussions

A number of themes were identified by authors’ iteration within and across the transcripts: (1) variation in perception of consanguineous unions and associated health risks, (2) the importance of informed choice and (3) preferences for information and sources of information (Fig. 1).

#### Theme 1: Variation in perception of consanguineous unions and associated health risks

Participants represented a variety of cultures, religions and ages, expressing a wide range of views on and experiences of consanguineous unions. This variation also informed participants’ views of health risk associated with consanguineous unions.

##### Subtheme 1.1: Variation in participants’ views on consanguineous unions

Participants’ overall views on consanguineous unions highlighted both the associated benefits and disadvantages, with views ranging from supportive to sceptical. Variations in opinion were marked by differences in religious beliefs and age. For example, a participant explained that consanguineous marriage is permitted in her religious beliefs and health risks are not a paramount concern. However, she was also aware of significant variation in opinion within the Islamic faith.

There are different religions, in Islam God did not forbid it. For my daughter, she will like her cousin, I will say go for it, it is not forbidden... Not all Muslim is the same, some say no. (Age 46).

The generational gap also appeared to divide opinions, with participants discussing that the older generation may show more support for consanguineous unions than the younger generation and that it is important to understand perspectives from difference generations.

New generation and old is completely different, so it is good to talk to both. The new generation, they are against this cousin marriage. (Age 46).

##### Subtheme 1.2: The role of personal experiences

Participants with experience of disability in children of consanguineous marriage were more aware of the potential health risks and were more critical of consanguineous unions, whilst participants with experience of consanguineous unions leading to healthy progeny or disability in progeny of unrelated parents believed that parentage was unjustly associated with disability. Many women detailed experiences within their own families, with a participant giving an account of her experience with physical disability in a child.

I knew [about the risks] before I came here because I saw it with my own eyes. A cousin married, and baby born with just one eye, it’s horrible... There is more chance [of disability] and I don’t like that. (Age 40).

Other participants considered the association between consanguineous unions and genetic disability to be over-emphasised. These participants cited cases where healthy children were born in consanguineous unions and children with disabilities were born in non-consanguineous unions. For example, a participant with experience of disability occurring a child of unrelated parents expressed her belief that consanguineous unions should not be associated with a definite risk of disability.

It is not a 100% chance a child will be ill. A cousin married outside [the family] and has autism in family. It’s a risk to take regardless. Need to change people’s views. (Age 64).

#### Theme 2: The importance of informed choice

Discussion of participants’ own experiences highlighted the importance of choice as well as the variety of factors that influence marriage decisions. Participants expressed a desire for more information on health risks and highlighted awareness in the context of informed choice.

##### Subtheme 2.1: Factors influencing choices in marriage

The factors thought to influence marriage decisions ranged from social and economic to religious and cultural. Motivations for marrying within one’s family included alignment in religious affiliation, preservation of traditional values, financial security, keeping close familial ties and protecting assets.

Community people like to talk and gossip about cousin marriages that are related to castes and inheritance. (Age 35).

At the same time, participants also discussed some of the benefits of marrying outside the family, including avoidance of family conflict, extending family networks and experiencing new cultures. For some women, autonomy in marriage decisions where love, happiness and choice were said to be paramount. One participant voiced their support of autonomy in marriage very clearly, stating:

Parents should not interfere and force cousin marriages, the people getting married should think for themselves. (Age 64).

##### Subtheme 2.2: Desire for information

Participants requested information on risks associated with consanguineous unions to enable educated decision-making, inform choices for their children and invest into the future, with most participants citing this as their motivation for joining the discussion.

Participants were generally aware of the link between consanguineous unions and illness, and were unclear on the nature of the association and which conditions had a genetic component. Many participants expressed difficulty understanding the mechanisms of Mendelian inheritance, leading to difficulties in comprehending genetic risk and the risk of disability that might be associated with consanguineous unions. The low risk of disability in children of both consanguineous and unrelated parents also contributed to difficulty in understanding the level of risk that is attributed to consanguineous unions.

I knew there’s a low risk of disability, I don’t know exactly how much. (Age 35)*.*

Discussions also reflected limited understanding around the genetic mechanisms behind disease and about which conditions arise from recessive genetic disorders. For example, one participant believed that infectious conditions, such as meningitis, were due to consanguineous unions but was corrected by another participant who explained that this was not the case and went on to elaborate that some disorders have complex aetiology and are not associates with genetics alone:

… meningitis does not come from internal marriage. Also, in UK there is many autistic children, maybe because of food they are eating or something around. [It] can be when women are pregnant. (Age 46)*.*

##### Subtheme 2.3: Awareness and educated decision-making

Most participants agreed that marriage decisions ultimately belong with the couple and advocated for increased awareness of the risks associated with consanguineous unions to promote educated decision-making. One participant clearly articulated this view by stating:

If there is higher genetic risk in cousin marriages then before getting married the two people could consider their genetics and so they can find out their genetic risk before getting married. (Age 35).

Another participant warned that whilst raising awareness and increasing community discussion is important, over-emphasis of the risks may generate anxiety for consanguineous couples and families:

Want to emphasise that it’s low risk, because you don’t want to scare all those that are already married within cousins but say the risk is there and just make sure they know. Also tell them about the genetic tests available if they like a cousin because not everyone knows. (Age 35).

Discussions reflected the belief that education on genetic risk is necessary to inform marriage choices. However, it was clear that other motivators for the marriage could take precedence despite knowledge of the risks associated:

Many people have their own purpose of cousin marriages and to fulfil the purpose they do not think about the long-term risks such as increased risk of genetic disease. (Age 35).

#### Theme 3: Preferences for information and recommended sources

In addition to variation in the perception of consanguineous unions and a desire for information to inform decision-making, preferences for the distribution of information about consanguineous unions was a key area of discussion.

##### Subtheme 3.1: Education with respect for health and social factors

Participants’ discussions highlighted the need for education to promote informed decision-making. Participants were widely accepting of question 8 of the topic guide (Table 1) which enquired about the acceptability of educating children on genetic literacy. The benefits of education included raising awareness, encouraging open discussion and passing information to future generations.

A course should be created to raise awareness about cousin marriage to inform people of the benefits and risks associated. This should be a life skill. (Age 61)*.*

Participants emphasised the importance of a universal approach in education which is culturally sensitive and considerate of that of the social factors associated with consanguineous unions. This universal approach was highlighted by a participant who stated:

Secondary school and college definitely it should be integrated with science not made a separate topic, so they don’t feel targeted. (Age 35)*.*

##### Subtheme 3.2: Accessible information

Participants identified the need for widespread dissemination of information about genetic risk through media which is readily accessible to the community, such as posters, advertisements and local media outlets. The use of printed media for information sharing, such as newspapers, posters and leaflets, was suggested for community spaces, religious centres and GP offices. Participants emphasised the value of an accessible approach, highlighting the need for local resources to be accessible to women and children in the community.

Local library is good. Family resource centre is really good … [you] can bring children there is small creche. It is important, majority of women cannot go to talk because of their children... The women would be learning for their children (Age 46).

Medical practitioners were also recognised for their role in disseminating information about health risks. However, participants acknowledged that medical guidance is often limited since discussions usually occur after a woman becomes pregnant, rather than during prenatal counselling. This highlights the need for a multi-faceted approach to information dissemination to ensure information is accessible.

Cousin marriage will only be raised when the wife is pregnant and at this stage it could be too late (Age 35)*.*

##### Subtheme 3.3: Information from influencers

Many women noted that authority figures may influence marriage decisions within their families, indicating a need to engage with community leaders, health professionals and religious leaders. Participants also considered discussions between parents and children as an important source of influence and opportunity for discussion, emphasising that education across multiple generations would be mutually reinforcing.

Parents with friendly children, the children can understand their parents. So, if you encourage parents with children, they can give the choice then to the children (Age 32).

Improving awareness in men was highlighted as an important area for improving community awareness. Participants felt that men had little engagement with the topic of consanguineous unions, even though they were important decision makers in marriage arrangements. Lack of engagement from men was considered a missed opportunity for increasing awareness, but one participant suggested this was changing due to education on the topic:

Men have different types of discussions about cousin marriages and they do not go into such detail. Educated men have more awareness of this than uneducated men … (Age 61).

## Discussion

### Emergent themes

Analysis of findings generated three emergent themes on consanguineous inions and the associated health risks. Variation in perception is a central theme, influencing the subsequent themes of informed choice and preferences for information. The varying opinions on consanguineous unions amongst participants can be explained by their diverse backgrounds and experiences (see Table 2). The discussions also highlighted a gap in genetic literacy which is reflected in our themes of variation and the importance of informed choice.

Participants made several recommendations for dissemination of information, emphasising the need for a multi-faceted approach. Parents and men in the community were also identified as potential influencers for spreading information. It is evident that future efforts to reach out to communities with health information about consanguineous unions should involve identification and engagement with the influencers with communities.

In light of participants’ acceptance of children receiving information about genetic risk, an educational intervention for genetic literacy holds potential for success in a diverse community, such as Newham. However, such an intervention must be carefully co-developed with community members to account for variation in views and the myriad of factors which play into marriage decisions, whilst avoiding stigmatisation of the community. The information requested by participants centred around the genetic mechanisms of disease and which conditions may be genetic in nature, highlighting a desire for improved genetic literacy. However, this study highlights the importance of recognising that consanguineous unions do not just present a simple “health risk”, but have wider social, economic and political dimensions in the complex context of marriage. Any service which is developed to address genetic literacy and enable informed choice must be respectful of the themes for variation in views and preferences for information, taking special care to avoid the stigma which was a concern for some participants.

Some participants felt consanguineous unions are over emphasised in their community, a finding which has been linked to alienation and stigma in previous studies (Ali et al. 2012; Ajaz et al. 2015). This suggests that efforts to improve genetic literacy should take a universal approach to avoid stigmatising a particular group and be informed through community engagement. Further to this, it became clear that the term “consanguinity” was novel to some participants. Many participants were aware of the term “cousin marriage” or “internal marriage”, but few reported awareness of the term “consanguinity” prior to the discussion group. Awareness of the terms used within communities will be vital for future engagement efforts.

### Findings in context

A large body of evidence supports an association between consanguineous unions and an increased risk of genetic disease (Bennett et al. 2002; Modell and Darr 2002; Strømme et al. 2009; Lyons et al. 2009; Shaw 2009; Shieh et al. 2012; Hamamy 2012; Darr et al. 2013; Ng 2016; Al-Mubarak 2017; Best et al. 2017; Sanyelbhaa et al. 2018). This association has become particularly concerning in the UK, with several studies focusing on consanguineous unions in Pakistani communities (Sanderson et al. 2006; Sheridan et al. 2013; Best et al. 2017). Some studies have focused on community perceptions of genetic risk (Ali et al. 2012; Ajaz et al. 2015; Darr 2016) and sought to inform interventions on how best to improve genetic literacy in consanguineous populations (Khan et al. 2016; Salway et al. 2016; Ali et al. 2018).

Previous research on perceptions of consanguineous unions support our findings that personal experiences may shape individual opinions on the risks associated with consanguineous unions, with some members of British Pakistani communities disputing the risks (Ajaz et al. 2015). The confusion around quantifying genetic risks in consanguineous unions is also a theme identified by several qualitative studies (Ajaz et al. 2015; Darr 2016). Our findings on services for genetic literacy also reflect those of similar research conducted in the UK and Netherlands, highlighting the risk of adding to perceptions to stigma in communities where consanguineous unions are common and reaffirming that health risks may not be the primary drivers in marriage decisions (Salway et al. 2016; Ali et al. 2018).

To our knowledge, this is the first study of women’s perception of consanguineous unions in a London Borough. This study provides unique insight into perceptions of consanguineous unions and genetic risk and indicates the acceptability of educational interventions to improve genetic literacy in children. Findings highlight the potential for co-design to navigate the variations in opinion throughout the community whilst addressing the desire to seek knowledge for informed choice, expanding on the findings of similar research on health literacy in the UK (Ali et al. 2018). This highlights the importance of co-design for developing services, increasing community awareness and making services and information accessible.

### Limitations

This study has several limitations, primarily due to biases in the self-selected study population. Our study sample was recruited by invitation from a community facilitator and is therefore likely to be composed of individuals who are interested in health outcomes in their community. These individuals may have a level of knowledge about consanguineous marriage which differs systematically from others in their community. This recruitment limitation is further illustrated by the fact that all participants were able to participate in the discussion groups in English, indicating that strong English speakers may have been more inclined to attend the discussion. The limited study population consisted of only women, with the majority being of Asian Pakistani background and 30 to 44 years of age (Table 2). Future qualitative research should aim to engage a wider demographic for data triangulation, including male participants and individuals of broader age ranges. Including men in future research should be a priority, since gender differences were highlighted in the present study and previously published literature (Buunk 2017).

The presentation at the start of the discussions was intended to start discussion but may have introduced some biases regarding awareness of genetic risk. A further limitation comes from the nature of groups discussions, whereby strong opinions from outspoken participants may overshadow the responses of others. This has been mitigated by the use of trained facilitators. Although the facilitators were trained to avoid leading questions and affirmative responses, reporter bias cannot be ruled out as a potential limitation due the reliance on assisted discussion. Furthermore, variable proficiency in English may have created barriers to discussion by some members, especially where conversation was fast paced or complex in detail. However, participants were aware of facilitators’ ability to translate into various languages if needed.

Despite some debate over the suitability of group discussions for exploring sensitive topic such as consanguineous unions, group discussions have proven efficacy in research on sensitive topics, including family planning and reproductive health, (Linhorst 2002; Van Teijlingen and Pitchforth 2006; Bowling 2014) and provide insight into community beliefs through their interactive nature (Gothberg et al. 2013). This methodology has proven success in understanding group perspectives, particularly around health issues, and can lead to improved candidness in responses when compared to individual interviews due to a perceived “safe space” and ability to build on ideas through discussion (Bowling 2014). We also did not collect details on participants’ marriage status or personal experience with consanguineous unions, which could have been useful in characterizing the influence of personal experience on their opinions.

## Conclusion

Overall, this study emphasises the need for awareness, educated decision-making and co-developing educational materials regarding consanguineous unions to support marginalised communities. Participants were widely receptive and engaged by the subject matter presented for discussion and requested additional community engagement.

## Electronic supplementary materia

ESM 1 (PDF 2589 kb)
