# Supplementary material for: Exploring perceptions of consanguineous unions with women from an East London community: analysis of discussion groups
Source: J Community Genet. 2019 Jul 16;11(2):225–34. doi: 10.1007/s12687-019-00429-4 (PMC7062959; doi:10.1007/s12687-019-00429-4)
Supplement: Supplementary file 1 — (PDF 2589 kb) [file 12687_2019_429_MOESM1_ESM.pdf]

[illegible]

awareness

desire for information

culture + family drivers

information based in fact/education

Transcript 1:

1. *To start, would you all like to share what has brought you here today?*

25: Her family has lots of problems because of cousin marriages. Has thalassemia in family and sight problems. She wants to know more information regarding this. Has 3 children, therefore want to know more. she wants to tackle the cultural aspect of this. She believes we should look at the medical facts. Has seen a lot of cousin marriages in family. Her family is too emotional and therefore falls into the cultural and family pressure of cousin marriages. She thinks we should plan for the future of her kids.

awareness due to family history.

desires information for children.

cultural/familial pressure?

education based on medical facts.

20: Believes that we need to be educated regarding this topic. Need to educate people about why the medical issues are occurring. Aware that disabilities occur and want to know if anything new is out there regarding this.

26: Life is important. She wants to plan for future generations. She wants to and believes we need to convey this information to our families so we can stop this.

desire for information to invest in future.

2a. What do you think of the presented information?

26: Couldn't hear the presentation properly. Lots of noise issues.

→ participants had some dificulty hearing the presentation.

20: Would have been better to hear the presentation a bit better. Would have been better if there was a mic. Would have been good to explain in different languages.

→ sitting according to background/language would improve reception of information.

25: People from same background in the same group sitting together would have been better.

2 b. Did you know about this topic before today?

25: Did not know about the term consanguinity.

26: Heard from Iram. Heard the term Asian marriages. Did not know it is about cousin marriages.

11: Never heard of the term consanguinity. But knew about cousin marriages.

10: Never heard of the term consanguinity. But knew about cousin marriages.

although participants were aware of cousin marriages, they had not heard the term consanguinity.

Group 1: People were not aware of consanguinity. But knew that cousin marriages occur.

2c. How would you describe the awareness if this topic in the community? How about between genders?

[illegible]

views on cousin marriages

is this contradictory?

25: People are not aware too much about it. People are ignoring the risks. People are ignoring the doctors advise. If people know the proper knowledge about it, if people knew the consequences about it then people might be more aware of risks.

10: The old generation people - they don't believe in medicine and therefore don't believe the information that is out there.

25: People believe that its destined from god and that these marriages were planned and destined by god. Lack of education. People are usually stubborn and don't want to believe that there are risks associated sometimes even if they are educated on this topic. It's better for mothers to have this information about consanguinity so they can change that and stop their children from going through any risks.

3a. what would you think about a family member marrying a cousin?

10: Her mum and dad are cousins and they believe that in cousin marriages, there is a lot of security and love and protection if cousins and therefore marry cousins.

11: One should have the knowledge about the health issues. In general, should be aware of the risk factors. There is a lack of knowledge regarding this. Her grandma asked her to marry one of her cousins. She doesn't want within the family. She doesn't want to be stuck in the same bubble. Cousin is like brother and sister relation to her and therefore doesn't want cousin marriages.

20: Believes that within cousin marriages, domestic violence may be involved. (family conflict)

3b. How would you describe the knowledge of consanguinity in your community?

Group 1: The word consanguinity needs to be more explored and explained what it is. People aren't aware of the health risks associated with cousin marriages. They are not aware that the risk is increased. Poor knowledge regarding this. We need to make people more aware of it.

4a. We are going to talk about the health problems that can be associated with consanguineous marriages. Before today, what had you been told about these risks?

11: Has seen enough examples out there regarding health issues.

20: In cultural point of view, it seems normal for cousins to get married. In Islam, it allows for cousins to get married and maybe that is why people ignore the health risks.

4b. What are couples entering a consanguineous marriage told about these health risks?

people have poor awareness and are ignoring the risks.
education will improve awareness
age and medical beliefs contribute to ignoring risks.
cultural beliefs play into views of marriage.
stubborn and don't want to change their beliefs
mothers need education.
cousin marriages offer love / protection
information about health issues. current lack of this.
grandmother wanted her to marry cousin.
doesn't want to be stuck.
harder to escape domestic violence.
improve awareness of term.
need to improve awareness of health risks.
poor knowledge associated.
awareness tied to seeing examples.
cultural norm - not topic of discussion in Islam.

Group 1: The vast majority are probably aware of it but they ignore it. And lot of people don't have the knowledge. They need medical professionals explaining it. Most of them are not told anything regarding the health risks as they do not see any professional regarding the cousin marriages. They usually only have their family members and older generation giving them advice.

awareness is there but ignored.

lack of medical knowledge.

poor awareness due to lack of professional consultations.

advice comes from family.

5a. What sources would you trust when learning about consanguinity?

Group 1: Medical professionals, Religious priests/religious people, doctors (GPs), health visitors, internet but depends on the source in internet and if it's a trusted website, from council meetings.

trusted sources of information.

5b. What do you think influences people's opinion on this topic?

Group 1: Most people are old school minded. To come out of that, needs a lot of education. Need to get out of the cultural and family pressure and be aware of the risk that they are putting their children in.
Knowing some facts about the health risks may help.

education needed to change mind-sets.

cultural and family pressures cloud awareness.

education improves awareness.

5c. What is the role of your local borough in providing information on consanguinity and health risks to children?

Group 1: This focus group is a good step forward, council should make effort to make more meetings like this. It shouldn't be a one meeting. It should be an ongoing project. Newham is an area where there is lot of cousin marriages. Lot of Asians in this community, therefore more facilities should be provided. Should have a big session regarding this event. Should have these topics talked about more as it is to do with people's health.

desire for more involvement by the council.

generating discussion will improve awareness

6a. imagine you are part of a team responsible for sharing information with the community in Newham, what approach would you take?

Group 1: Would put up information and posters in these following places: local GPs, children centres, community centres, gatherings, health centre, mosques and religious places, Library, pregnant ladies classes and sessions, Schools and bus stops. Would provide facts in these posters and also provide contact numbers for anyone who would like to discuss this further and so that people are aware that support is out there. At GPs. There should be posters available in community. For example, how they have for smoking. Also highlight this issue in newspaper and monthly magazine (Newham magazine), Newham recorders (local newspaper)– the main issue raised should health risk. Having focus groups should be advertised.

methods to improve awareness

educational focus should be health risks.

6b. What are the key messages that need to be highlighted in the community?

Group 1: Health factors. Risks that are involved in cousin marriages. Risks should be highlighted.

health risks are most important focus.

6c. Who is the most important target for this information?

target for awareness.

Group 1: Everyone should be targeted. Asian population of all ages. This will help to avoid the health problems and therefore help the next generations.

universal approach to improving awareness.

6d. What will people think of this information?

Group 1: People will be willing to receive knowledge. Children should be more aware. People are more open minded and therefore will be more open to listening. There will still be people who will take in a negative way as it still happens in their families. People will be offended. But information needs to be give out.

open to ed.

awareness in children.

views tied to family experience

7a. Do you think this information should be available to the wider community? Why do you think this? Who do you see as the target for this?

Group 1: Everyone should be targeted- it should be of all age- more in Asian background.

universal awareness.

8. What do you think of children learning about consanguinity and genetics in school?

Group 1: They should be taught in secondary school and colleges. About the risks and health issues and general awareness about it.

education on health risks on secondary schools / college.

9. Of everything that we discussed today, what do you think is the most important?

Group 1: Educating the community and the health risks. Main concern is the health risk. Lack of knowledge can itself lead to health risk.

focus on education of health risks.

Transcript 2:

1. To start, would you all like to share what has brought you here today?

6: To gain information, for my children.

desire for information for children.

18: Aunt and uncle are cousin-marriage, and has disabled cousin. So she was motivated to have more information and learn more.

awareness due to family history.

family history contributing to desire for information.

2a. What would you think about a family member marrying a cousin?

30: There are different religions, in islam, god did not forbid it, but he did forbid marriage to brother, father, brother of my father, brother of mother. This is not allowed. It is incest. Brother's uncle is also not allowed. Apart from those, everything else is allowed. The son of my uncle, he is older than me, no problem. I am allowed to marry him. Because god he said that, I do not think there is a problem. If they say no it is not god, you will get disabled children. Maybe scientifically it is true – but it is not haram. For my daughter, she will

religous beliefs don't discuss health risks.

views based on religion.

acknowledgement of medical / scientific risks, not backed by religion

↓

disconnect?

differences in family views - some may promote it and others prevent it. ↔ variation in family views.

like her cousin, I will say go for it, it is not forbidden. If he is meant to be disabled, that is for god. Not all muslim is the same, some say no.

6: Some family don't like it when you marry out of family, especially muslim.

30: Maybe she likes her cousin, she knows him, she will be in love with him, she will marry him. Some parents they will forbid it even if she says she likes him. Then we create problem, they can run away, it will be bad because there is not consent from the father and problems then start. It is not blessed, it is not haram. If one of the daughter like the cousin, it is up to them.

18: They don't like marriage in the cousin – this new generation they know if they marry cousin there will be disabled children. My sister, her child is disabled, but she has beautiful three boys. Still married but mostly the cousin say they do not want to marry cousin, they have the awareness.

5: They know they have the risk of medical disability, autism and disabled.

13: Child asked her: if I marry my sister it is better because then I keep her close by and she will not move away. But she explained to child that you cannot marry sister, but then he asked why can I not marry sister, can I marry cousin.

has medical knowledge and rejected cousin marriage.

30: Bengali, studying medicine in 3rd year. His father wanted him to marry his cousin, the boy declined and has not returned to the house again. Too many problems. The parents cannot force the children.

Iram: Traditionally we follow our forefathers, my granddad before he passed, my son's daughter is going to marry my daughter's son, after children grow up they have different views, but traditionally we would follow the will of the grandfather's wishes sine he is now passed. But now the elders' wishes do not have the same importance to the youngers. But the family is close, we know them better than strangers. Maybe they have big land and property, they don't want the son in law to marry daughter and coming to split the land. In cosmopolitan city, it is different. But it remains rural traditions. Children are more educated now.

5: There is still some conflict in families.

?: There remains the caste issue also. Will marry my daughter within the caste. If you go for other clan/tribe/caste they are different – different thoughts and backgrounds.

38: Same experience in Pakistan – agrees with I story.

13: With newer arrived families, they still have the same ideas.

6: It is about knowing the family and the background.

would support daughters choice. Religon suggests this is predetermined. variation in beliefs between muslims.

some families prefer cousin marriages.

some families may prevent cousin marriage + create familial conflict.

younger generation has more (-) outlook.

family experience.

family knowledge contributing to awareness.

awareness of risk in younger generation.

view on consanguinity to keep families together.

parents cannot force decision, leads to (-) relationship.

younger generation rely less on family opinons. Land/wealth may be driver for decisions.

conflict in family.

caste/socio-economics play into marriage choices.

families from different immigration histories have different views.

views depend on knowing family - familiarity effects views.

30: Some children educated now will ask why, they will know of the problems. Better to marriage cousins, better than to bring strangers. My sister lives back home, she wishes that my son to marry her daughter. I am against, but it is not for disabled, but because my sister will stay my sister, but if there is problem in the marriage, then I will lose my sister. I know people, their children marry Algerian, they go and then they need just papers and then they go back and leave them. So it is better to avoid, it is better to avoid marriage with the strangers. But children these days they will not follow you. And you cannot force them, there is nothing you can do. The children are educated, they are independent.

* education contributing to altered views in children.
view family as better option than strangers.
sister wants children to marry.
views (-) because doesn't want to ruin relationship.
cannot force children.
education in children.

6: In my family, first cousins married. But there is many problems with asthma and allergies. It is to do with the marriage. (My daughter is 1year old, has same problem, but my husband not cousin. So children are healthy when one parent is out of family. Otherwise every month many problems, many hospital time.)

(6) aware that asthma not genetic?

this reads odd - daughter is ill but states children are healthy with no cousin marriage?

* awareness due to family experience

38: Asthma is quite common, I don't think it is from internal marriage. My mum has asthma, but she is not from blood relation parents, just that in this country, many people have asthma.

knowledge of conditions which are associated to cousin marriage.

5: I know someone married to a cousin, she had 3 children autistic. One died of meningitis. One is normal.

30: But meningitis does not come form internal marriage. Also in UK, US there is many autistic children, maybe because of food they are eating or something around. Can be when women are pregnant, many people in England they have children with autism and when they pregnant they had depression, anxiety, other problems. Maybe this is why the children have autism. Meningitis does not come from cousin marriage.

debate over which conditions are genetically associated due to consanguinity.

2c. How would you describe the knowledge of consanguinity in your community?

23: Yes we have to clarify the medical problem from cousin marriage. It is not clear. There is someone who has a child, cannot keep salt, always tired. Hammersmith hospital said maybe it is because you are cousin marriage, this problem was explained. There are many things coming up from cousin marriage - disability, autism…but we cannot say what will definitely happen, we do not know our father, forefathers and so on, what was the medical problem then. It is not clear which is from the cousin marriage. Are issues discussed within the family?

desire for clear information.
can't be certain of outcome from cousin marriage.

30: It is about education. Some are ignorant, this is how they think and you cannot change their mind. The son daughter to marry the family, not to go outside. Depends on the people how they are.

awareness depends of education.

23: But it is better to discuss in the family. To know the family background. But you have to share the news, that it might happen.

discuss risks within family.

30: My husband, he used to live with his parents in Algeria. His mother father want him to marry the daughter of his aunty. They wanted him to marry her, but he said no he did not like her, so he went to study in France, then his mother always say you must stay in contact. He knew me there before, he was happy. When we go back to Algeria with our children, he says thanks god. But he knows his mother tried to force him, but she understood, she saw her son very happy, maybe if he marry that girl he would not be happy. She has no regret. It depends on the family, the parent.

family experience of rejecting cousin marriage with (+) outcome. happiness.

23: If you force your children to marry their cousin, they may say we will do it, but after when they have problems, they will blame their parents – that is very common. It is because of you I am not happy.

forcing children to marry will lead to resentment.

30's daughter arrived (is #41 hereafter) – asked what she thinks about cousin marriage

41: There is nothing wrong with cousin marriage, but it is not for me personally.

daughter of participant speaking of her views openly. Does not favour cousin marriage.

30: I would promote the cousin, you know him, he speaks the same language, but before we would have to obey the parents wishes, but no longer like that.

mother of 41 → would promote cousin marriage but respects daughter's choices.

34: My sister and cousin have marriage – they have four children, the younger son is disabled, his eyes, cousin marriage is many many problem.

awareness of risk due to family experience.

What if your husband or father felt differently from you? Would it be different if they felt in a different way to you?

23: Depends on your husband and how understanding he is. My son is 8, my husband already wants someone from back home for him, but I told him no, we don't know her, how she grew up, her education, cousin marriage has some family issues, yes she is my brothers daughter, but if something not good, then I don't want it to be between me and my brother. Wait until they grow up and chose their own way.

does not want cousin marriage for her son due to not knowing her.

worry over conflict between siblings if children's marriage fails.

Do you think its mostly women thinking about these issues, the risks and illness?

3: I think men are more busy in their routine work, they go there, come back from food. The day is done, the women have to look after and cleaning. They (women) met people at school and meetings so they have the time to go GP and social network, so they are more aware. Men are very restricted. Just food TV and sleep.

women have more awareness than men.

5a. What sources would you trust when learning about consanguinity?

30: Local library is good. Family resource centre is really good. Some people go there, there is coffee, can bring children there is small crèche. It is important, majority of women cannot go to talk because of the children. The

local resources which are accessible to women with children.

crèche is useful when available. the women would be learning for their children.

[resources in social spaces.]

6: Conversation café, coffee morning. Community centre.

8: After school. But it is hard to say.

3: Better through their mothers.

41: Mothers are good because they will give honest opinions, and they will know of people in the community who have certain experiences.

[education throug[illegible] mothers and social connection[illegible]]

5c. In your opinion, what is the role of your local borough in providing information on consanguinity and health risks to children

[local resources]

30: The borough is good – the family resource centre.

6. Imagine that you are part of the team responsible for sharing information with the community in Newham.

[social / accessible sources of information.]

3: Arrange coffee morning

30: Leaflets and through newham magazine. So long as it is confidential. New generation and old is completely different. So it is good to talk to both. The new generation they are against this cousin marriage. Maybe the older people think it is better, we know them.

[divide between generations.]

8: Parents with friendly children, the children can understand their parents. So if you encourage parents with children, they can give the choice then to the children.

[educated parents lead to greater educated choice in childre[illegible]]

6: The family will talk about the girls, even when they kids, they will see the girls and

23: There is more pressure from family to marry when you are certain age.

[children pressured to marry at certain age.]

8: When 12 to 13 years back home, tey looking like ladies already. People ask for the daughter to be given to the son, so there is much pressure from family to marry. People who want to come from home will hope for the marriage too. Actually many children do not like to go back home because they are worried they will be married away.

[pressure from relatives at home
↓
children avoid this pressure.]

30: In the future they will not have problem. With experience. One asian to marry one African – is different culture, many people marry and there is different food – husband wants spicy food, but the wife cannot cook. The mother in law she wants the wife to cook and clean, but they know different – they end up splitting. Some marry the same culture, it is easier. You like the same.

[views on cousin marriage and benefits of sharing culture.]

41: For most parents, culture is important. Parents want marriage within the culture.

30: I guide them, but if they like someone, what can I say.

23: Talking to children in a friendly way is the best, you cannot force them. You talk with them and give them the choice.

Do you think you are unusual for the community since you are here – down the high street would people have similar views?

23: Yes, it is the same. Many people must feel the same way.

30: Some people they are shy, they cannot talk open. They prefer to speak only in closed area. Everyone has their own opinion. One is naughty, she says she will not marry Algerian, she prefer English. These man are bossy, you cook for them. But English are open minded and different.

18: It is good to talk with everybody – good to hear the different opinions.

6: Men should come. Most important is education, after education it is a long life.

18: Men's is totally different. It is not hard, he is open minded, understands everything. Just he always send children: first education then marriage, then you have choice and if you like it, if not, leave to something else. That is our thinking, but we guide them this is right and wrong.

23: It is good to come and share the views, nice to hear the different experiences and share knowledge.

5: Good to know the different opinions, what they thinking and what they have shown in their community.

24: Good talking and having different opinions and good time to meet. I have attended many meetings but today is totally different.

6: Thank you, you have provided great information.

18: Yes we have really enjoyed it.

24: Good for the future of my children and to have different knowledge.

Transcript 3:

1. Would you like to share what has brought you here?

15: She wants to know information so she can think about her future including her children's' marriages.

parents want marriage in culture.

can only guide children.

children's choice

believes others share her views on choice.

shy - barrier to awareness.

variation in views.

prefers english men.

discussing different opinions.

need to educate men.

men are open minded and understanding.

guide who? children/men.

sharing knowledge.

sharing views.

different views/opinions.

shared information "great".

gain knowledge for children.

information for future and children.

32: The topic is interesting, she gets to give her own opinion and get information

14: Wants to know more information about cousin marriages.

32: Wants to know about the services in the borough to raise awareness about cousin marriages and how they work.

7: Wants to discuss cousin marriages and determine if it is beneficial or not. All her grandparents and the generations above her have married in the family. The rule in her culture (being Sri Lankan culture) is that the women can only marry their mother's brother's son and this is due to their understanding of genetics. They investigate first whether the woman or the man has any health issues and if there are none then they get married.

19: To find out any more research and information about cousin marriages.

4: To see if cousin marriage is bad or not

32: Is not against cousin marriage as she believes it is religiously correct. From her experience, she has seen that disease is not related to cousin marriage as many of her family have married in cousins and no health problems have appeared in their children.

33: She is married to her cousin and consanguinity is a very old practice.

2a. What do you think of the presented information?

7: Feels that genes are commonly 'blamed' and that it is an issue with cousin marriages that is excessively emphasised. She feels that people of Asian background come with a genetic disease is it instantly associated with consanguinity if present, but this does not occur in other ethnic backgrounds.

16: Says in Islam there is nothing against cousin marriages and the risk of genetic disease in cousin marriages is not mentioned in the Quran or the Prophet's sayings.

15: The increased of genetic disease in cousin marriages in not true and it is all about the fate of an individual. A genetic disease may not necessarily be due to a cousin marriage as it could have other medical causes such as a deficiency.

14: Her parents are cousins and there is no genetic condition in her family associated with cousin marriages.

7: The way of thinking about cousin marriage was different back in the days (in previous generations), today, in the 21st century it is more about love marriage and cousins can fall in love and get married. Many cousin marriages are for property and inheritance purposes.

share opinon, get information.

more information.

information on services.

definitive information on risk.

family and culture has rules about cousin marriage.

research.

definitive information.

religion supports cousin marriage.

not against cousin marriage due to religion and experience.

old practice.

genes blamed and over emphasis in Asian ethnicity.

religion does not tell of risks.

fate, genetic risk not true.

genetic risks due to other causes.

no genetic disease in family.

shift in younger gen toward love marriage.

also marry for other property reasons.

contradicts itself - genetic risk due to "other causes" but also "fate"?

16: The family traditions and cultures are changing now, so reason for cousin marriages is changing.

traditions and ↓culture changing.
views changing.

2b. Did you know about this topic before today?

4: Yes, she briefly heard of the risks associated, her mum wanted her to marry her cousin. It is about a person's point of view and perception.

heard of risks.

15: She heard of the risks from different people she knows.

heard of risks.

7: She had heard of the risks associated with cousin marriages as she took part in nurse training in Germany. Many people have their own purpose of cousin marriages and to fulfil the purpose they do not think about the long-term risks such as increased risk of genetic disease.

awareness due to medical training
acknowledgement of ~~risk~~ different factors.

2c. How would you describe the awareness of this topic within the community? How about between genders?

7: In the past and back home, men were dominant, the decision makers, education wise and intellectually women were more thoughtful. In her opinion men cannot multi task, so they were working and earning money for their family there were not thinking about family and social matters. Men have less awareness of consanguinity problems and are not accepting of them.

men are decision makers.
men less aware

4: Not sure how aware men are on this matter compared to women. Men do not talk about these things so she doesn't know how aware they are.

men don't talk about this.

32: Men have different type of discussions about cousin marriages and they do not go into such detail. Educated men have more awareness of this than uneducated men.

educated men are more aware

7: Men agree that cousin marriages that lead to offspring with genetic diseases can cause a problem. She believes that men's opinion and level of information on consanguinity depends on how women such as their wives or sisters or mothers explain it to them, so they rely on others to increase their understanding.

men aware of genetic disease.
men's under-standing depends on women.

4: Increased family issues also occur in cousin marriages.

increased family issues.

7: Women should help men to understand. There needs to be an organisation to raise awareness in men.

need to raise awareness in men.

32: It is also about men's' interest in the topic and most men are too busy in work life to learn and pay attention.

men are disinterested.

7: If figures with authority or who are perceived as authoritative such as doctors and the local council, social workers etc, could help raise awareness maybe men will show interest and understand better.

authority figures to educate men.

33: Men in her family take more interest in family matters so the culture is changing and men are paying attention to social matters like consanguinity, so men should be continuously educated and awareness raised in both male and females.

men becoming more involved.

raise awareness for men and women.

3a. What would you think about a family member marrying a cousin?

7: If the person loves his/her cousin, then they should get married.

marry for love.

4: No matter what in laws say, ignore external opinions and what everyone thinks. Communicate with your cousin and get to know him further if you plan on marrying him as this is important too.

ignore opinons of others and communicate with partner.

32: She would ask the person who is marrying their cousin if they are happy with it and if they know the person well.

happy / know partner.

7: If there is a higher genetic risk in cousin marriages then before getting married the two people could consider their genetics and so they can find out their genetic risk before getting married.

be aware / educated on genetic risk.

19: She feels that she would not get involved and should not give her opinion of someone she knows is marrying their cousin but she would think about the reason behind the consanguineous marriage.

not her business, many reasons behind marriage.

33: She thinks the person marrying their cousin needs to be 100% sure what they are doing and the reason for marrying their cousin and think about their marriage.

think about reason behind marriage.

3b. How would you describe the knowledge of consanguinity in your community?

7: Cousin marriage will only be raised when the wife is pregnant and at this stage it could be too late.

awareness raised too late.

32: She heard on Islam channel on TV that in Malaysia, people must do a course before getting married.

education before marriage.

7: She said the course in Malaysia in only for ethnic minorities there and not Malaysians.

education focused on minorities.

32: A course should be created to raise awareness about cousin marriage to inform people of the benefits and risks associated. This should be a life skill

weighing risks and benefits in marriage should be a life skill.

19: Only GPs and doctors talk about consanguinity when it is relevant (the patient has already married their cousin)

awareness raised too late.

7: Community people like to talk and gossip about cousin marriages that are related to castes and inheritance. Families can propose cousin marriage since the child is born and he/ she can be forced to marry their cousin. 8 generations

marriage for £

choice by family / forced?

of participants 7's family has been married in cousins however careful selection of cousins is involved to minimise the health risks. She found out about the health risks associated with consanguinity through her nursing placement and other people in her expertise and uneducated people are not aware of the health risks associated. She has experience of German cousin marriages and Arab cousin marriages and in only one family she found a child of a consanguineous marriage had autism. This made her think that the risk of genetic disease in a consanguineous marriage is quite low.

32: Participants sister got married in South Africa and she was told by her doctor that she should not marry her cousin due to the risks, her dad was also told this but did not think about it. She and her sister (who both married their cousins in the same family) both have disabled children but do not think it is due to marrying their cousin. The GP does not about the patient's future decisions so may feel that he cannot discuss consanguinity with them.

5a. What sources would you trust when learning about consanguinity?

7: Registrar officers should be trusted with information about consanguinity. In Germany, they ask about the relationship between the husband and wife before marriage and if they are cousins a consent form must be signed to show awareness of the risks.

6. How should young people or any other audience be informed about consanguinity?

4: Children centre should raise awareness to mothers and children on cousin marriages.

7: Young children will not understand cousin marriage issues. GPs should raise awareness.

32: Registrar raising awareness may be too late to raise awareness as the man and woman are ready to marry.

33: Genetic testing could be beneficial to raise awareness about consanguinity and minimise the health risks.

32: We need to know both sides being the communities and the side of the people raising awareness about it. Everyone should know the pros and cons of cousin marriages and be open minded as it is not about not marrying your cousin at all. A survey should be conducted about cousin marriages and the percentage that are successful, socially successful as well and health wise.

What would make cousin marriages a success?

32: Had an arranged marriage with her cousin

33: Cousins getting married should discuss as they are the ones getting married. They should also think about the risks of the marriage.

discuss risks in marriage.

32 & 33: Are sisters married into the same family as they lost their mother at a young age and so their father got them married to their cousins in the same family so they could be together and be well cared for

marriage to keep family together.

7: Arranged cousin marriages could turn into love marriages

become love marriage.

4: Had an arranged marriage with her cousin and it turned to a love marriage.

become love marriage.

7: Cousin marriages also have a greater chance of domestic violence as cousins know each other better from before.

risk of domestic violence

7a. Who should be targeted when making this information available to the wider community?

32: Children should be educated on consanguinity at around 18 years old. Some information should also come from social services and awareness should be raised about the risks of a consanguineous marriage in secondary school.

education in secondary school to raise awareness

7: Both men and women should be educated on cousin marriages and the age to raise awareness about this should be 14 years old and above.

educate @ 14

32: There should be subjects in school about on consanguinity and marriage in general as well as respecting spouses and this should come under health education. It is more important to educate children than adults as they should be aware before it's too late. → concept from why GPs and registrars are ineffective.

health education should include consan.

8. What do you think about children learning about consanguinity and genetics in school?

7: All children should be educated in cousin marriages aged 14 and above. 32,33 ,4 and 19 all agreed to this.

educate @ 14

7: Cousin marriages problems can also involve family politics.

family politics.

★ 19: The problems with cousin marriages are not just health related, there are also family politics issues. Consanguinity should not be reduced to a health issue but is more of a social issue.

not only health

teach as social issue

7: She conducted a questionnaire when she was in Germany and her results found that children of cousin marriages from the maternal side have increased disability. She wants more detailed research done on the genetic health risk associated with consanguinity.

more research on risk (quantify)

19: Most Asian communities around the world have cousin marriages. Some people avoid cousin marriages because if there is a problem in the couple this

leads to family feuds and family breaking. This is a reason to not marry in cousins.

risk of family conflict.

33: Parents should not interfere and force cousin marriages, the people getting married should think for themselves.

parents should not interfere.

9. Of everything that we discussed today, what do you think is the most important?

7: Educating everyone on consanguinity and giving them important information about it like the health risks

education on health risks.

4: Finding out the background of the person their marrying whether it's their cousin or not.

awareness of relation to spouse.

32: Consider a person upbringing and back ground and be open minded when informing people about consanguinity so raise awareness accordingly.

be mindful of backgrounds when considering consan.

33: Parents should not force and interfere with a couple's marriage and the couple should think and get to know each other.

parents should not interefere.

19: People should get a chance to talk and know each other properly both socially and medically, this will help reduce the genetic risk and other problems.

awareness of individual and health risks must be promoted to mitigate range of complications.

32: Even in a cousin marriage there should be trust and honesty and faithfulness. People should be educated before reaching the marriage point in life, with regards to cousin marriage, people should be educated beforehand.

social values.

educated before.

Transcript 4:

1. To start, would you all like to share what has brought you here today?

12: Iram told me to come. Didn't want to come at first as they married cousin. Just to show its normal they wanted to come. Rejected a cousin marriage on mum's side first after going through with the nikkah. Guy from Canada, didn't want to move but family were saying NO. Family were saying its already done so you have to do it. I couldn't handle it, broke it off, family upset, so it caused family problems, relationship affected within family. Next married family member from dad side so caused conflicts between mum and dad side.

family conflicts due to politics of cousin marriage.

31: See and hear lots of experiences. Understands the risks and other than that, find the other side that is that it causes many problems with the extended family. Breaks families. As an outsider looking in, I also see the benefits and advantages.

family conflict.

both good/bad.

8: Didn't want to come because of stigma of marrying cousin, has a son that has autism but doesn't believe it's due to marrying cousin. She fancied him so her choice, not forced.

stigma.

doesn't believe autism linked to son's.

It's not like its 100% child will be ill. A Cousin has married outside and has autism in family. It's a Risk to take regardless. Need to change people's view.

view due to experience.

37: Sister in law side saw so many disabled children, 1 in 4 some sort of mental problem. Due to marriage within family. I am against it.

view due to experience.

28: New experience, see peoples view on it. If you're in love with cousin different story, you can't stop it but in general if you're forced then I'm against it.

personal choice.

36: The topic is interesting when Iram told me. Family all married within family besides me. Everyone is the same, the culture is the same. I feel left out. They always get together. It's a community. Seen lots of diseases that kids have- friends with disability. Majority within family.

family community with consan.

2b. Did you know about this topic before today?

12: Family experience so know enough. General understanding, maybe not that much about the science

general but not scientific understanding

8: Knew a lot about of it.

aware.

28: Knew a lot.

aware.

Group 4: General consensus that everyone knew a lot about it.

2c. How would you describe the awareness of this topic in the community? How about between genders?

8: Community know about it, they know everything we know

Community has awareness

36: Everyone, in Muslim community, has awareness but doesn't make much difference. Hindus don't because something about they're not allowed, again with Sikhs, black community again don't care- they don't have much of an understanding compared to us Muslims.

awareness but

35: In afghan community very common. Say it's good and no problem.

12: Muslim community all know, not sure about Hindu

9: Again all Asians know about this.

17: Feel like everyone knows about it, my family, lots are married within, for example my cousin have two disabled children, one of them died at 9 year olds, they know about the consequences before the death but they still do it. I think in Punjab it's the culture, they have to marry forcefully

know risks and ignore.

cultural pressure.

28: In my community they believe to marry within family some with different opinion, I always preferred outside the family, no fighting peace and quiet. 2

more peace without consan.

of my sisters have, and so much problems. I don't like it. Just choose someone different. My parent's opinion, they think it's good but not me, I don't think it's good. Be healthy, be happy, out of family

negative perception due to experience of family conflict.

37: Communities know less, this generation knowledge is better à know it's risky à less people do it, plus lots of genetic tests are available and lots of people do it and stop marrying cousin if genes will cause a problem

better awareness in younger gen.
awareness due to genetic testing.

3a. What would you think about a family member marrying a cousin?

8: Absolutely fine as long as they're happy, I care about my kids being happy, if they want to marry within go ahead, if not go ahead marry whoever, they're happy I'm happy

happiness in marriage is most important.

9: Good for out of family, forcing they think it's like a brother and sister married.

forced marriage leads to poor perception.

17: If they're happy then they're fine, it's up to them

happiness.

12: As long as they're not forced I have no problem about it

choice.

36: I think for a girl it is easy to adapt, their thinking is the same, she knows the tradition she can fit in easily. She knows the expectation. I married outside the family, but all my siblings have married inside, I see them altogether doing things. I feel like they have less changes to enhance their knowledge because they're being brought up with same thinking people so they're not exposed to other ways of thinking.

marriage within family limits new experiences.

37: I don't like it, I don't force my children. They can choose. You can't extend your family, with strangers you can extend your family and have a big family and I like that.

extend family.

35: No I don't like the idea

negative view

4a. Before today, what had you been told about these risks?

8: There's a risk of autism

know risk

12: I knew there's a low risk of disability, I don't know exactly how much

low risk,
how much?

37: I knew before I came here, because I saw with my own eyes, a cousin married and baby born with just one eye, it's horrible. After I saw that, I never will push my children, I will tell them about this case and its unhealthy. There's more chance and I don't like that if you see the consequence it's better not to do that they continue to marry just cousin regardless, they are rich don't want to share money to outsiders. Not educated, not enough knowledge.

negative perception due to experience.

lack of aware- due to lack of knowledge.

36: Every time I get pregnant they tell me about risks even though I haven't married family. I used to research that's how I know all.

28: Big risks, I've got thalassemia, my RBC gets low but my husband has no problem so our children are safe.

awareness of own risks.

4b. What are couples entering a consanguineous marriage told about these health risks in your community?

8: No who is going to tell them? No one emphasises on the risks

no one to educate/ spread awareness.

12: People wouldn't say it to anyone else. Not their business, they will not want to interfere, it's their choice

personal choice.

37: Job of parents, no one outside in community will say that and get involved.

parents should educate.

38: Parents can only, if we have the knowledge we can tell our child

parents must be educated.

28: Most of them they don't

9: New generations know about the test, they can tell their children

younger know about genetic testing.

35: I feel like they are told about it in the community.

awareness in community.

17: No I don't feel like they're told about the risk

no awareness.

trust info from doctors... however it comes too late

5a. What sources would you trust when learning about consanguinity?

8: Medical and experiences from what I've seen,

doctors/experienc

28: NHS doctors

doctors

9: Medical professions

doctors

36: Personal experiences and doctors

doctors/experienc

37: You can research as well to find about it, NHS TRUST, from experiences

research/ doctors/ experience.

12: Medical professionals

doctors

17: We can google it. I feel like in communities there will be lots of opinions, not all will be facts, I will trust doctors.

online

35: NHS and doctors

doctors.

6a. What approach would you take?

12: Workshops and focus groups like this. Make leaflets especially in GP's reception, so they can hand it out. Put posters up – hospital/dentist/GP/primary school will give info to mothers so they can pass to children and educate. Also, universities, so they can be aware as they want

information in public spaces for women/ mothers.

to get married. Make a very basic leaflet with pro and cons. Go to colleges and university.

awareness in college campuses.

36: I think proper medical advice is needed. In schools, teachers will be respected so they need to be told, they can advise the future generations.

education from respected sources

8: I would educate the parents more than children

educate parents

17: Leaflets hand out to the community

awareness in leaflets.

9: I think men need to be aware. I think school especially secondary. Also, community works, like group awareness. Its like sex education, they need to know about this too. Secondary school is good to target and tell them. If they know can give to parents.

men need more awareness

educate in social / school like sex ed. (health ed)

37: College is the most important place to give information to, they need to be targeted to spread out the information.

educate in college

35: Secondary school, teenagers they need to know

educate in secondary school.

6b. What are the key messages that need to be highlighted in the community?

8: Highlight the mental and physical issues. I think in Newham there's a rise of illnesses and I think that's what has made parents fed up, so if they're made aware they will understand why there's this increase in such conditions.

highlight health risks.

17: Highlight disability

highlight health risks.

12: Want to emphasise it's a low risk, because you don't want to scare all those that are already married within cousins but say the risk is there and just make sure they know. Also tell them about the genetic tests available if they like a cousin because not everyone knows.

emphasis on low risk and sensitivity.

genetic tests.

9: Mental and physical disabilities. From personal experience I've seen this and I want to make this aware.

highlight health risks

awareness from personal exp.

37: Mental and physical disabilities

highlight health risks.

6c. Who is the most important target for this information?

12: Students need to be educated and made aware and also parents because they're ignorant, both need to be targeted. Education needed on both side

students / parents

9: Parents need to be made aware

parents

8: Parents need to be told, because children are knowing it and they're fighting their children so the parents need to know

parents

17: Parents, again same as everyone else

parents.

37 & 35: Seminar and conference to tell parents so if they know it and also to students in school. Both need to know consequences, so parents can't force the children and children if they know enough they don't do that as well.

parents.

parents allow choice.

7. Do you think this information should be available to the wider community? Why do you think this?

8: Definitely, because it is still happening. People need to know.

need awareness.

9: More community know about it more awareness

community awareness.

12: In terms of wider community, in terms of diverse group, independent white/black/sikh community, it doesn't happen, mainly Asian groups so target them. Focus groups etc to these target group. Focus on the Asians, identify the communities and target them, no point wasting resource.

targeted approach.

17: Should be available to everyone.

everyone.

37: Spread out all this info, in communities' town hall, give that info as much as you can, when that knowledge goes up, they know that risks. I like that they did today, came out and made aware to us, I think this is the best approach.

spread information for knowledge and risk awareness.

35: Spread to wider community.

wider community

8. What do you think of children learning about consanguinity and genetics in school?

12: Secondary school and college definitely
it should be integrated with science not made a separate topic so they don't feel targeted

integrated into science curriculum.

8: Agree as above.

35: Secondary definitely

secondary

37: Secondary

secondary

17: Secondary school

secondary

9. Of everything that we discussed today, what do you think is the most important?

12: Making people aware of it, education. Increases own awareness

education vital for awareness.

8: Nice hearing peoples' opinion and experience, also the genetic testing I didn't know about that so that was very important for me. I didn't really understand it before but talking now I know more.

nice to hear experiences of others.

37: Awareness, the consequences, everyone here knows now so they can spread.

aware of risks

9: Again, hearing about everyone's experiences, it's nice.

nice to hear experiences of others

35: I learned the consequences, I have a 12 years old daughter and now I can give her info. I have a cousin in Germany who has asked for my daughter and I'm like no no. so I can tell her.

awareness/ info for daughter.
